# Neuroimaging Correlates of Depression—Implications to Clinical Practice

**DOI:** 10.3389/fpsyt.2019.00703

**Published:** 2019-10-01

**Authors:** Lígia Castanheira, Carlos Silva, Elie Cheniaux, Diogo Telles-Correia

**Affiliations:** ^1^Departamento de Psiquiatria, Faculdade de Medicina, Universidade de Lisboa, Lisbon, Portugal; ^2^Clínica Universitária de Psicologia e Psiquiatria, Faculdade de Medicina, Universidade de Lisboa, Lisbon, Portugal; ^3^Instituto de Psiquiatria da Universidade Federal do Rio de Janeiro (IPUB/UFRJ) & Faculdade de Ciências Médicas da Universidade do Estado do Rio de Janeiro (FCM/UERJ), Rio de Janeiro, Brazil

**Keywords:** major depressive disorder, major depression, magnetic resonance imaging, positron emission tomography, neuroimaging

## Abstract

The growth of the literature about neuroimaging of major depressive disorder (MDD) over the last several decades has contributed to the progress in recognizing precise brain areas, networks, and neurotransmitter processes related to depression. However, there are still doubts about the etiology and pathophysiology of depression that need answering. The authors did a nonsystematic review of the literature using PubMed database, with the following search terms: “major depressive disorder,” “neuroimaging,” “functional imaging,” “magnetic resonance imaging,” “functional magnetic resonance imaging,” and “structural imaging,” being selected the significant articles published on the topic. Anterior cingulate cortex, hippocampus, orbitomedial prefrontal cortex, amygdala basal ganglia, and the cerebellum were the main affected areas across the selected studies. These areas respond to particular neurotransmitter systems, neurochemicals, hormones, and other signal proteins; even more, the evidence supports a distorted frontolimbic mood regulatory pathway in MDD patients. Despite the positive findings, translation to treatment of MDD remains illusory. In conclusion, this article aims to be a critical review of the neuroimaging correlates of depression in clinical research with the purpose to improve clinical practice.

## Introduction

Major depressive disorder (MDD) remains a critical disease that greatly impacts the global burden of disease (1). In the absence of biological markers, clinical-based methods continued to be the gold standard to diagnose this disorder (2). In clinical assessment, a nosological classification is used, according to international systems such as the *Diagnostic and Statistical Manual of Mental Disorders, Fifth Edition* (3) and the *International Classification of Diseases, 10th Revision* (4). Efforts are continuously made in order to discover dependable biomarkers that clarify the neurobiological mechanisms of psychiatric disorders, identify populations at risk, and provide etiology-based treatments (5).

Studies involving imaging modalities such as structural magnetic resonance imaging (sMRI) and functional magnetic resonance imaging (fMRI) aim to outline brain irregularities accompanying MDD. Moreover, the knowledge provided by the neurobiological components resulting in the pathogenesis of MDD can explain the existence of biomarkers for diagnosis, prognosis, and response prediction (5).

In this article, we expect to review the neuroimaging correlates of depression in clinical research. These neuroimaging correlates of depression are presented and discussed from a critical perspective.

## Methods

The authors performed a nonsystematic review of the literature using PubMed database, with the following search terms: “major depressive disorder,” “neuroimaging,” “functional imaging,” “magnetic resonance imaging,” “functional magnetic resonance imaging,” and “structural imaging.” The literature search was limited to the English language and limited to the dates up to January 2019. There were selected 28 significant articles published on the topic.

## Results

### Neuroimaging Modalities in Depression

**Table 1** contains the 26 articles that were selected and are divided by the type of neuroimaging modality and by brain regions studied in the respective article. One article used positron emission tomography (PET) and sMRI modalities, and the other were developed using only one modality. The imaging technic more focused on was fMRI (13 articles), followed by PET (eight articles) and sMRI (four articles).

**Table 1:** Neuroimaging modalities in depression.

Rank	Article	Neuroimaging modality	Main brain areas studied
1	Deep brain stimulation for treatment-resistant depression (6)	PET	Subgenual cingulate region
2	Resting-state functional connectivity in major depression: abnormally increased contributions from subgenual cingulate cortex and thalamus (7)	fMRI resting state (RS)	Subgenual cingulate region, thalamus
3	Subgenual prefrontal cortex abnormalities in mood disorders (8)	PET sMRI	Subgenual prefrontal cortex
4	Hippocampal atrophy in recurrent major depression (9)	sMRI	Hippocampus
5	Resting-state functional MRI in depression unmasks increased connectivity between networks *via* the dorsal nexus (10)	fMRI-RS	Cognitive control, the default mode, and affective networks
6	The default-mode network and self-referential processes in depression (11)	fMRI task based (TB)	Default-mode network
7	Depression duration, but not age, predicts hippocampal volume loss in medically healthy women with recurrent major depression (12)	sMRI	Hippocampus, amygdala
8	Failure to regulate: counterproductive recruitment of top-down prefrontal-subcortical circuitry in major depression (13)	fMRI-TB	Prefrontal cortex, amygdala
9	Untreated depression and hippocampal volume loss (14)	sMRI	Hippocampus
10	Subcallosal cingulate gyrus deep brain stimulation for treatment-resistant depression (15)	PET	Limbic and cortical regions
11	Deep brain stimulation to reward circuitry alleviates anhedonia in refractory major depression (16)	PET	Frontostriatal networks
12	Increased amygdala and decreased dorsolateral prefrontal BOLD responses in unipolar depression: related and independent features (17)	fMRI-TB	Amygdala, dorsolateral prefrontal cortex
13	Increased amygdala response to masked emotional faces in depressed subjects resolves with antidepressant treatment: an fMRI study (18)	fMRI-TB	Amygdala
14	Identifying major depression using whole-brain functional connectivity: a multivariate pattern analysis (19)	fMRI-RS	
15	Nucleus accumbens deep brain stimulation decreases ratings of depression and anxiety in treatment-resistant depression (20)	PET	Subgenual cingulate, prefrontal regions
16	Disrupted brain connectivity networks in drug-naive, first-episode MDD (21)	fMRI-RS	
17	Role of translocator protein density, a marker of neuroinflammation, in the brain during major depressive episodes (22)	PET	Prefrontal cortex, anterior cingulate cortex, insula
18	Evidence of a dissociation pattern in resting-state default-mode network connectivity in first-episode, treatment-naive major depression patients. (23)	fMRI-RS	Anterior medial cortex, posterior medial cortex
19	Attenuation of the neural response to sad faces in major depression by antidepressant treatment: a prospective, event-related functional magnetic resonance imaging study (24)	fMRI-TB	Amygdala, striatum, frontoparietal cortex, pregenual cingulate cortex
20	Reduced prefrontal glutamate/glutamine and gamma-aminobutyric acid levels in major depression determined using proton magnetic resonance spectroscopy (MRS) (25)	MRS	Dorsomedial and dorsal anterolateral prefrontal cortices
21	Default-mode and task-positive network activity in MDD: implications for adaptive and maladaptive rumination (26)	fMRI-RS	Default-mode network, task-positive network, right frontoinsular cortex
22	A functional anatomical study of unipolar depression (27)	PET	Prefrontal cortex, amygdala
23	Can’t shake that feeling: event-related fMRI assessment of sustained amygdala activity in response to emotional information in depressed individuals	fMRI-TBw	Amygdala
24	Cingulate function in depression: a potential predictor of treatment response (28)	PET	Rostral anterior cingulate region
25	Toward a neuroimaging treatment selection biomarker for MDD (29)	PET	Insula
26	A differential pattern of neural response toward sad versus happy facia expressions in MDD (30)	fMRI-TB	

### Functional Magnetic Resonance Imaging

#### Resting-State Functional Magnetic Resonance Imaging

Major depressive disorder is characterized by depressed mood, anhedonia, and feelings of worthlessness; some of these alterations are related to the self, such as rumination (31–33) and autobiographical memory (34). Functional neuroimaging, in this case, fMRI, has achieved to isolate brain regions implicated in self-relation, for example, the anterior cingulate cortex (ACC), the medial prefrontal cortex (MPFC), the posterior cingulate cortex (PCC), dorsomedial thalamus, and the precuneus (35, 36).

The default-mode network (DMN) is composed by the lateral and medial parietal cortex, ventral and dorsal medial prefrontal cortices, and areas of the medial and lateral temporal cortices, (11) and is thought to be responsible for processing information related to survival instinct, as well as the capacity to plan the future, desires, and beliefs; all these tasks are related to the self (37–39), functions that are intertwined with the self.

Among the articles cited in fMRI, two demonstrated that subjects with MDD were prone to have an increased activity in the MPFC/ACC areas, as well as diminished activity in the PCC/precuneus and bilateral angular gyrus areas (10, 23).

The cognitive control network (CCN) is an entity responsible for attention-demanding cognitive tasks (40). The affective network (AN) is composed of regions of the ACC (41) responsible for processing emotions (41–46) and is crucial in fear, vigilance, and other emotional responses (43).

One of the articles selected showed increased connectivity in depression in the bilateral dorsomedial prefrontal cortex, which encompasses the DMN, AN, and CCN (10). Greicius et al. (47) show increased functional network connectivity in the thalamus, subgenual cingulate, the precuneus, and the orbitofrontal cortex (OFC) (7). The other two articles demonstrated a significant alteration in global brain networks focusing mainly in the DMN area and in the AN (19, 21).

Regarding effective connectivity of different brain areas, different from functional connectivity, effective connectivity is the effect one neuronal network employs on another network (48). Used an unusual method to measure that connectivity; they used spectral dynamic causal modeling (spDCM) (48). Using spDCM, found a decreased influence from the anterior insula to the middle frontal gyrus in medicated subjects with MDD. An important nexus between the anterior insula and amygdala was also found. A positive correlation between hippocampal node activation and the severity of depression was found, which confirms the relation that the right anterior insula has on depression pathophysiology (48). There was also a meaningful interconnection with activation in the right superior parietal lobule, the right precentral and postcentral gyrus, and in left precuneus (48, 49).

#### Task-Based Functional Magnetic Resonance Imaging

In depression, emotions tend to be perceived and processed erroneously; for example, good events tend to be assimilated and processed as negative or harmful to the person involved in them (50–54). It is also known that population with MDD has difficulty in recognizing and processing emotion in facial expression (sad vs. happy). The brain networks related to the identification of emotional facial expressions are the fusiform area in the ventral occipitotemporal cortex (55–57); the superior temporal sulcus (58); and the amygdala.

Sheline et al. (18) demonstrated in depressed patients a greater activation of the left amygdala during early stages. Besides, they also confirmed that after 8 weeks of antidepressant treatment (with selective serotonin reuptake inhibitor–sertraline 100 mg/d) amygdala activation decreased drastically (18).

Fu et al. (59) found that presentation of sad faces led to increased activation of the left hippocampus and mainly the amygdala and parahippocampal gyrus. They also reported the activation of the thalamus, dorsal cingulate gyrus, hypothalamus, ventral striatum, and insula. They also found that treatment with fluoxetine 20 mg/d led to a reduction of the response in the ventral striatum and thalamus (24).

In fMRI-TB, it is also possible to register disturbances in the DMN, Sheline et al. (11) report increased activity in the DMN in depressed subjects. In some proposed tasks, subjects would maintain or increase DMN activity, while control subjects would diminish DMN activity (11).

Translation is the dominion where it is possible to shift data across different subjects to enrich and perfect diagnosis and subsequent treatment, keeping in mind applicability in daily clinical decisions. In psychiatry, it is much more complicated because of the heterogeneity and variability of clinical symptoms; for example, it is highly subjective to measure reliably the level of sadness or anhedonia in two or more people who suffer from MDD, which led to the necessity of devising a hypothesis of translation in depression (60). Stoyanov and colleagues (60) conceptualized an approach of translational cross-validation of psychiatric neurocognitive tests (Von Zerssen’s Depression scale) with fMRI scans, expecting to find associations between these methods. Stoyanov and colleagues (61) operationalized this concept, and in early findings, there is a weak correlation between the medial frontal cortex (MFC) and MDD subjects; activation in anterior thalamus, hippocampus, and parahippocampal gyrus, areas implicated in the pathophysiology of MDD, was reported as well.

### Positron Emission Tomography

In PET, we are able to estimate brain functional degree on a regional scale. This is possible by quantifying the emission of positrons due to the half-life decay of the various radiopharmaceuticals. Different isotypes permit to evaluate different neurotransmitters receptors, hence its versatility (62).

Relating to the subgenual cingulate region, three articles revealed decreased brain activity/metabolism in patients with depression (8, 20, 28). Mayberg et al. (63) demonstrated that it was possible to diminish the intensity of depressive symptoms, in resistant MDD, through electrical stimulation of the subgenual cingulate white matter (6). Relating to limbic and cortical regions, including frontostriatal networks, two articles revealed altered metabolic activity in those areas after deep brain stimulation of the subcallosal cingulate gyrus/nucleus accumbens in subjects with refractory depressive disease (15, 16).

Two articles demonstrated increased microglial activation in patients with depression and identified that response to escitalopram or behavior therapy was predicted by insula activity level (22, 29).

### Structural Magnetic Resonance Imaging

Four of the articles that evaluated hippocampal volume identified a volume reduction in patients with MDD (9, 12, 14, 64). Hippocampal volume reduction is observable in MDD (65, 66), mainly in the first episode (67). The cognitive decline observed in MDD over the various episodes of illness may be due to volume decrease in the hippocampus (68). Treatment with antidepressants may revert neurocognitive symptoms due to hippocampal volume increase (69). Arnone et al. (70) found that treatment with citalopram led to hippocampal volume increase after 8 weeks of treatment.

Kandilarova, (71) studying the volume of gray matter in affective disorders, found a decrease in gray matter volume, specifically in MDD. The main cluster affected (reduced gray matter) was the MFC and the ACC. The other relevant region was the OFC. Gray matter reduction in the ACC is probably related to the abnormalities found in cognitive and affective regulation, attention, problem solving, motivation, and decision making. Furthermore, the decrease in OFC gray matter explains the alterations in social and emotional behaviors and also in the processing of reward and punishment (71). Kong et al. (72) studied the impact of treatment of MDD with fluoxetine and reported volume increase mainly in the orbitofrontal and the dorsolateral cortices.

Sheline et al. (12) examined the amygdala, with a decreased bilateral amygdala core nuclei volume in patients with recurrent depression. Drevets et al. (8) demonstrated a subgenual prefrontal cortical volume reduction in subjects with depression.

### Magnetic Resonance Spectroscopy

Shen et al. (25) through MRS examined the dorsomedial and dorsal anterolateral prefrontal cortices and concluded that in depressed patients the levels of glutamate, glutamine, and γ-aminobutyric acid were decreased.

## Conclusion

In this article, we reviewed the neuroimaging correlates of depression, in various imaging modalities such as PET, MRI, fMRI, and MRS.

According to the studies we have reviewed, MDD influences major brain areas such as the DMN, AN, CCN, and amygdala and that these affected areas respond to medication, antidepressants.

When evaluating structural differences in brain areas in MDD, we find different variations through multiple brain regions. Nonetheless, evidence has been found that supports changes in gray matter volume in cortical and subcortical regions that might be associated with depressive states. These changes are present through the course of the illness.

The concept of translation is applied globally in medicine, except in psychiatry, mainly due to the heterogeneity of clinical symptoms. Small steps have been made in the field of translational neuroimaging. Nevertheless, they are promising. Despite advances in research on the genetic neuroimaging, psychoneuroimmunology, and multimodal imaging, further studies are necessary to confirm this concept in all these modalities, diminishing the gap between neuroscience and clinical psychiatry.

A significant weakness of the reviewed studies is that they generally have a small population, so we have to be careful in drawing conclusions to the general population. In addition, different studies focus on different brain areas, not being able to identify a pathognomonic finding of MDD. We also fail to conclude whether such differences represent a congenital structural anomaly, a result of the disease, or a compensatory adaptation.

The tendency to evaluate particular brain regions independently is an explicit limitation, as the various areas are interrelated. The circuit-based analysis will provide a foundation for behavioral process analysis. This will facilitate the identification and analysis of MDD and psychiatric symptoms, mostly subjective, but that is used in clinical practice.

More studies, with larger populations, and ideally focusing on circuits other than specific brain areas, will be necessary to draw further conclusions.

Studies including subjects not medicated might enlighten about MDD-related brain abnormalities, without possible unwanted effects that the medication might introduce.

## Author Contributions

All authors of this study had an active role in the manuscript and have thoroughly read the final manuscript.

## Conflict of Interest

The authors declare that the research was conducted in the absence of any commercial or financial relationships that could be construed as a potential conflict of interest.

The handling editor declared a past co-authorship with one of the authors DT-C.
